# Characteristics of Health Care Organizations Associated With Clinician Trust: Results From the Healthy Work Place Study

**DOI:** 10.1001/jamanetworkopen.2019.6201

**Published:** 2019-06-21

**Authors:** Mark Linzer, Sara Poplau, Kriti Prasad, Dhruv Khullar, Roger Brown, Anita Varkey, Steven Yale, Ellie Grossman, Eric Williams, Christine Sinsky

**Affiliations:** 1 Hennepin Healthcare Research Institute, Hennepin Healthcare, University of Minnesota, Minneapolis; 2 Cornell University Medical Center, New York, New York; 3 University of Wisconsin School of Nursing, Madison; 4 Loyola University School of Medicine, Chicago, Illinois; 5 University of Central Florida College of Medicine, Lake Nona, Orlando; 6 Cambridge Health Alliance, Cambridge, Massachusetts; 7 Culverhouse College of Business, University of Alabama, Tuscaloosa; 8 American Medical Association, Chicago, Illinois

## Abstract

**Importance:**

There is new emphasis on clinician trust in health care organizations but little empirical data about the association of trust with clinician satisfaction and retention.

**Objective:**

To examine organizational characteristics associated with trust.

**Design, Setting, and Participants:**

This prospective cohort study uses data collected from 2012 to 2014 from 34 primary care practices employing physicians (family medicine and general internal medicine) and advanced practice clinicians (nurse practitioners and physician assistants) in the upper Midwest and East Coast of the United States as part of the Healthy Work Place randomized clinical trial. Analyses were performed from 2015 to 2016.

**Main Outcomes and Measures:**

Clinician trust was measured using a 5-item scale, including belonging, loyalty, safety focus, sense of trust, and responsibility to clinicians in need (range, 1-4, with 1 indicating low and 4 indicating high; Cronbach α = 0.77). Other metrics included work control, work atmosphere (calm to chaotic), organizational culture (cohesiveness, emphases on quality and communication, and values alignment; range, 1-4, with 1 indicating low and 4 indicating high), and clinician stress (range, 1-5, with 1 indicating low and 5 indicating high), satisfaction (range, 1-5, with 1 indicating low and 4 indicating high), burnout (range, 1-5, with 1 indicating no burnout and 5 indicating very high feeling of burnout), and intention to leave (range, 1-5, with 1 indicating no intention to leave and 5 indicating definite intention to leave). Analyses included 2-level hierarchical modeling controlling for age, sex, specialty, and clinician type. Cohen *d* effect sizes (ESs) were considered small at 0.20, moderate at 0.50, and large at 0.80 or more.

**Results:**

The study included 165 clinicians (mean [SD] age, 47.3 [9.2] years; 86 [52.1%] women). Of these, 143 (87.7%) were physicians and 22 (13.3%) were advanced practice clinicians; 105 clinicians (63.6%) worked in family medicine, and 60 clinicians (36.4%) worked in internal medicine. Compared with clinicians with low levels of trust, clinicians who reported high levels of trust had higher mean (SD) scores for work control (2.49 [0.52] vs 2.18 [0.45]; *P* < .001), cohesiveness (3.11 [0.46] vs 2.51 [0.51]; *P* < .001), emphasis on quality vs productivity (3.12 [0.48] vs 2.58 [0.41]; *P* < .001), emphasis on communication (3.39 [0.41] vs 3.01 [0.44]; *P* < .001), and values alignment (2.61 [0.59] vs 2.12 [0.52]; *P* < .001). Men were more likely than women to express loyalty (ES, 0.35; 95% CI, 0.05-0.66; *P* = .02) and high trust (ES, 0.31; 95% CI, 0.01-0.62; *P* = .04). Compared with clinicians with low trust at baseline, clinicians with high trust at baseline had a higher mean (SD) satisfaction score (3.99 [0.08] vs 3.51 [0.07]; *P* < .001; ES, 0.70; 95% CI, 0.39-1.02). Compared with clinicians in whom trust declined or remained low, clinicians with improved or stable high trust reported higher mean (SD) satisfaction (4.01 [0.07] vs 3.43 [0.06]; *P* < .001; ES, 0.98; 95% CI, 0.66-1.31) and lower stress (3.21 [0.09] vs 3.53 [0.09]; *P* = .02; ES, −0.39; 95% CI, −0.70 to −0.08) scores and had approximately half the odds of intending to leave (odds ratio, 0.481; 95% CI, 0.241-0.957; *P* = .04).

**Conclusions and Relevance:**

Addressing low levels of trust by improving work control and emphasizing quality, cohesion, communication, and values may improve clinician satisfaction, stress, and retention.

## Key Points

**Question:**

Which organizational characteristics and clinician outcomes are associated with clinician trust in the organization?

**Findings:**

In this cohort study of 165 clinicians from 34 primary care clinics as part of the Healthy Work Place study of work life–related interventions, high trust was associated with higher self-reported sense of work control, cohesion, emphases on quality vs productivity and communication, and values alignment with leadership. Trust was associated with clinician satisfaction; increased trust through time was associated with more satisfaction, less stress, and greater plans to stay with the practice.

**Meaning:**

Focused improvement of organizational characteristics may build clinician trust and be associated with clinician satisfaction and retention.

## Teaser

This cohort study uses data from the US Healthy Work Place study to examine characteristics of health care organizations that are associated with clinician trust in the organization.

## Introduction

While trust has long been understood as vital for the patient-clinician relationship,^1,2^ less attention has been given to the degree to which physicians and other clinicians trust the organizations in which they work and the association of trust or distrust with organizational performance and patient outcomes. This is starting to change amid increasing recognition of stress associated with work and professional dissatisfaction among physicians^3,4,5,6^ as well as a policy shift toward measuring the performance of health care organizations.^7,8,9,10^

Physician burnout within health care systems is being addressed at the national level,^11,12,13^ but to our knowledge, few recent studies have explicitly evaluated the associations of clinician trust and organizational characteristics with clinician outcomes, such as satisfaction, stress, burnout, or intent to leave. Trust as a key organizational characteristic may be associated with burnout, but they are not the same construct: clinicians may lack trust in their organization or feel their values do not align with those of their leaders without feeling burned out. To our knowledge, the literature on physician trust tends to be theoretical, not empirical.^14^ Research from management and nursing literatures suggests that organizational trust is an important element to a well-functioning organization,^15,16,17,18,19,20,21^ but the extent to which health care organizations benefit from or can modulate clinician trust is a gap in the literature, to our knowledge.

A 2009 study^22^ established associations of work conditions (eg, chaos, lack of control, time pressure) and clinician outcomes with patient outcomes (ie, quality and safety). A 2000 study^23^ found sex differences in several components of work life and wellness. In this study, we used the previously published conceptual model^22,23^ of the association of work conditions with clinician outcomes to examine characteristics within health care organizations associated with clinician trust, explore adverse clinician outcomes associated with lower trust, and determine if outcomes, including satisfaction, stress, burnout, and intention to leave, improve when trust improves. We used trust in the organization as an organizational culture variable to measure trust and sought to determine associations of trust building and sex differences with trust. Multilevel regression analyses controlling for key covariates were performed to test for these associations and outcomes. Prior studies (eg, the Minimizing Error, Maximizing Outcome study^22^ and the Physician Worklife Study^24^) guided the selection of the contributors, covariates, and outcome variables.

## Methods

### Study Sample

The Healthy Work Place (HWP) study,^11^ from which the data for our study were drawn, has been described in detail in the literature. The HWP study^11^ was a longitudinal, cluster randomized clinical trial in 34 primary care clinics with 168 clinicians (ie, general internists, family physicians, and advanced practice clinicians [APCs], including nurse practitioners and physician assistants) in the upper Midwest and East Coast of the United States. Clinicians were eligible for participation in the study if they had been with the practice at a minimum of 0.5 full-time–equivalent employment for at least 1 year. Site-specific institutional review board approval was obtained. Written informed consent was obtained from all participants.

### Study Design

Our study is a prospective evaluation using data collected as part of the HWP study.^11^ The HWP study was based on the conceptual model proposed by and refined after the Minimizing Error, Maximizing Outcome study^22^ found an association of work conditions with clinician and patient outcomes. Of the 34 clinics randomized to intervention and control arms, 17 underwent interventions, such as workflow redesign or chronic disease management programs, to address clinician stress and burnout.^11,25^

Work-life perceptions, including chaos in the workplace, organizational culture, and time pressure during office visits, and clinician outcomes (ie, satisfaction, stress, burnout, and intent to leave) were measured at baseline. (These data were reported in 2-page summary sheets only to the 17 intervention sites.) Most metrics were developed and validated from the Minimizing Error, Maximizing Outcome study.^22^ Organizational culture scales were adapted from studies by Curoe et al^26^ and Kralewski et al^27^ of medical group practices. After 12 to 18 months, follow-up data were obtained from all 34 clinics. For our study, data from all 34 practices in the intervention and control arms were combined. Data were collected from 2012 to 2014, and analyses were conducted from 2015 to 2016.

Trust in the organization, our primary outcome metric, was 1 of 5 organizational culture domains, which also included cohesiveness, an emphasis on quality vs productivity, an emphasis on communication and information, and values alignment between clinicians and their leaders. Each domain was scored on a scale of 1 to 4, with 1 indicating low and 4 indicating high. The trust variable consisted of 5 questions: sense of belonging, loyalty to the organization, responsibility to help clinicians with problems, safety culture (eg, reporting adverse events), and the degree to which clinicians trust their organization. Scores ranged from 1 to 4, with 1 indicating low and 4 indicating high. Trust was measured as the mean response to this set of 5 uniform-polarity items on a 4-point scale; as in the study by 2009 Linzer et al,^22^ high trust was defined as a mean score of 3 or more.

Clinicians were instructed to use their own definition of burnout to rank their feelings on a scale of 1 to 5, with 1 indicating “I enjoy my work. I have no symptoms of burnout”; 2, “Occasionally, I am under stress, and I don’t always have as much energy as I once did, but I don’t feel burned out;” 3, “I am definitely burning out and have one or more symptoms of burnout, such as physical and emotional exhaustion”; 4, “The symptoms of burnout that I’m experiencing won’t go away. I think about frustrations at work a lot”; and 5, “I feel completely burned out and often wonder if I can go on. I am at the point where I may need some changes or may need to seek some sort of help.” Intent to leave was measured by response to the question, “What is the likelihood that you will leave your current practice within TWO YEARS?” on a scale of 1 to 5, with 1 indicating none and 5 indicating definitely.

This study was performed according to the Strengthening the Reporting of Observational Studies in Epidemiology (STROBE) reporting guideline. Specific issues cited in the STROBE guidelines which are explicitly addressed in our study include sample size calculations (performed to achieve 80% power in the HWP study,^11^ although recruitment was somewhat short of full-sample estimates), missing data (multiple imputation methods were used), multiplicity (multiple comparisons were not corrected for, as we wished not to miss any variables that might be associated with trust), and generalizability (which was reasonable for the study, as multiple types of clinics [academic, inner city, suburban, and rural] were included).

### Statistical Analysis

Internal consistency of scales was assessed with Cronbach α statistic. Descriptive statistics were assessed using χ^2^, Fisher exact, and *t* tests. *P* values were 2-tailed, and statistical significance was set at less than .05. Cohen *d* effect sizes (ESs) were computed to provide estimates of the degree of difference between groups or change at different points in time. By standard convention, an ES of 0.20 was considered small; ES of 0.50, moderate; and ES of 0.80 or more, large.

We then constructed 4 multilevel models (to account for nonindependence of clinician observation nested in clinics). Two multilevel mixed-effects linear regression models were constructed to assess associations of perceived trust with continuous scales of stress and satisfaction, and 2 multilevel mixed-effects ordered logistic regression models were constructed for the ordered categorical items of burnout and intent to leave. We used these models to assess associations of clinician trust with outcome measures at baseline. We then used these models to assess associations of changes in trust with outcomes at the conclusion of the study. All models were adjusted for covariates of age, sex, type of practice (ie, family medicine vs general internal medicine), and clinician type (physician vs APC). Two main factors regarding trust were explored, including high vs low perceived trust at baseline and change in trust during the study, with changes defined as improved trust for clinicians who either improved trust scores or had high scores that remained high; clinicians with decreased trust were those with decreased trust scores during the study or with low trust scores that remained stable. Initial models were constructed for the 4 outcome variables, satisfaction, stress, burnout, and intent to leave, at baseline and as a function of change in trust. These models were then reconstructed by sex, with separate models (adjusted for the same covariates) for women and men. Continuous models using *R*^2^ were developed using the methods of Snijders and Bosker.^28^ Pseudo *R*^2^, constructed for categorical outcomes using the method of McKelvey and Zavoina^29^ with both fixed and random effects, allowed estimates of percentage of variance explained by the model.^29^ All analyses met model assumptions and were constructed using Stata statistical software version 15 (StataCorp).

## Results

### Baseline Characteristics and Trust Variable

There were 168 clinicians in the full HWP study,^11^ and 165 clinicians (mean [SD] age, 47.3 [9.2] years; 86 [52.1%] women), including 143 physicians and 22 APCs, had full data available for this study; 14 participants (8.5%) were lost to follow-up. Characteristics of the clinicians, separated into high vs low trust groups, are described in Table 1. Cronbach α for the full 5-item trust variable was 0.77 (satisfaction: α = 0.85; stress: α = 0.78; work control: α = 0.86; and values alignment: α = 0.83). Cronbach α more than 0.70 is considered acceptable for psychometric reliability. High trust was more commonly reported by men compared with women (43 men [54.4%] vs 35 women [40.7%]), family medicine clinicians compared with general internal medicine clinicians (53 clinicians [50.5%] vs 25 clinicians [41.7%]), and physicians compared with APCs (71 physicians [49.7%] vs 7 APCs [31.8%]). These differences were not statistically significant.

**Table 1.** Characteristics of Clinicians in the Healthy Work Place Study by Trust at Baseline

Characteristic	Low Trust	High Trust	*P* Value
Women, No. (%)	51 (59.3)	35 (40.7)	.08
Age, mean (SD), y	47.1 (8.7)	47.4 (9.6)	.80
Role, No. (%)			
Physicians	72 (50.4)	71 (49.7)	.11
Advanced practice clinicians	15 (68.2)	7 (31.8)	
Specialty, No. (%)			
General internal medicine	35 (58.3)	25 (41.7)	.27
Family medicine	52 (49.5)	53 (50.5)	
Work conditions score, mean (SD)			
Control	2.18 (0.45)	2.49 (0.52)	<.001
Pace	3.56 (0.70)	3.42 (0.65)	.22
Culture score, mean (SD)			
Cohesiveness	2.51 (0.51)	3.11 (0.46)	<.001
Emphasis on quality vs productivity	2.58 (0.41)	3.12 (0.48)	<.001
Emphasis on communication	3.01 (0.44)	3.39 (0.41)	<.001
Values alignment with leadership	2.12 (0.52)	2.61 (0.59)	<.001

Participants with high trust had a higher mean (SD) work control score compared with those with low trust (2.49 [0.52] vs 2.18 [0.45]; *P* < .001). Compared with participants with low trust scores, participants with high trust also had higher mean (SD) scores on organizational culture variables, including cohesion (3.11 [0.46] vs 2.51 [0.51]; *P* < .001), emphasis on quality vs productivity (3.12 [0.48] vs 2.58 [0.41]; *P* < .001), emphasis on communication and information (3.39 [0.41] vs 3.01 [0.44]; *P* < .001), and values alignment between clinicians and leaders (2.61 [0.59] vs 2.12 [0.52]; *P* < .001) (Table 1). Compared with men, women had a lower perceived sense of loyalty to the organization (mean [SD] score, 2.60 [0.81] vs 2.86 [0.65]; *P* = .02; ES, 0.35; 95% CI, 0.05-0.66), and a lower perceived degree of organizational trust (mean [SD] score, 2.23 [0.82] vs 2.49 [0.84]; *P* = .04; ES, 0.31; 95% CI, 0.01-0.62) (Table 2).

**Table 2.** Scores on Components of Trust Variables by Sex^a^

Statement	Score, Mean (SD)		*P* Value	Effect Size (95% CI)
	Men	Women		
There is a strong sense of belonging to the group	3.06 (0.85)	2.98 (0.94)	.59	0.09 (−0.21 to 0.39)
There is a great deal of organizational loyalty	2.86 (0.65)	2.60 (0.81)	.02	0.35 (0.05 to 0.66)
There is a strong sense of responsibility to help our physicians if they have personal problems	3.08 (0.85)	3.03 (0.87)	.68	0.06 (−0.24 to 0.36)
We encourage reporting of adverse patient events	2.98 (0.85)	2.92 (0.85)	.65	0.07 (−0.23 to 0.37)
There is a high degree of organizational trust	2.49 (0.84)	2.23 (0.82)	.04	0.31 (0.01 to 0.62)

^a^ Items scored from 1 to 4, with 1 indicating strongly disagree and 4, strongly agree. Cronbach α for trust scale (all 5 items) = 0.77.

### Trust and Its Association With Other Work-Life Metrics

Table 3 shows the multilevel mixed-effects regression model of the associations of clinician outcomes with trust. While all outcomes were more favorable in the high-trust group, only mean (SE) satisfaction score was significantly higher in clinicians with high trust compared with those with low trust (3.99 [0.08] vs 3.51 [0.07]; *P* < .001; ES, 0.70; 95% CI, 0.39-1.02). There were 66 clinicians in the stable high or increased trust group and 64 in the stable low or decreased trust group. The mean (SE) satisfaction score was substantially higher in clinicians in whom trust increased vs those in whom trust decreased (4.01 [0.07] vs 3.43 [0.06]; *P* < .001; ES, 0.98; 95% CI, 0.66-1.31). The mean (SE) stress score was significantly lower in clinicians with improved trust vs those with decreased trust (3.21 [0.09] vs 3.53 [0.09]; *P* = .02; ES, −0.39; 95% CI, −0.70 to −0.08). The full models, including contributors, covariates, and *R*^2^, are presented in eTable 1 and eTable 2 in the Supplement. After adjusting for covariates, change in trust explained 23% of variance in satisfaction score and 9% of variance in stress score.

**Table 3.** Work Conditions by Trust at Baseline and Change in Trust or Stable Trust Through Time^a^

Group	Satisfaction Score			Stress Score		
	Mean (SE)	*P* Value	Effect Size (95% CI)^b^	Mean (SE)	*P* Value	Effect Size (95% CI)^b^
Trust at baseline						
High (n = 78)	3.99 (0.08)	<.001	0.70 (0.39 to 1.02)	3.29 (0.08)	.16	−0.22 (−0.53 to 0.09)
Low (n = 87)	3.51 (0.07)			3.45 (0.08)		
Change in trust						
Increase (n = 66)^c^	4.01 (0.07)	<.001	0.98 (0.66 to 1.31)	3.21 (0.09)	.02	−0.39 (−0.70 to −0.08)
Decrease (n = 64)^d^	3.43 (0.06)			3.53 (0.09)		

^a^ Two-level hierarchical modeling controlling for age, sex, clinician type, and discipline (family medicine vs internal medicine).

^b^ Effect sizes: low = 0.20; moderate = 0.50; large ≥ 0.80.

^c^ Includes clinicians whose trust increased or stayed high through time.

^d^ Includes clinicians whose trust decreased or stayed low through time.

### Association of Trust With Burnout and Intent to Leave

Results of the multilevel mixed-effect ordered logit models indicated that the scores for burnout and intent to leave were lower (more favorable) in the increased trust subgroup compared with the decreased trust subgroup, although these differences were not statistically significant, with the exception of intention to leave; clinicians with high trust had half the odds of intending to leave (odds ratio, 0.481; 95% CI, 0.241-0.957; *P* = .04) (Figure). The full models for the Figure are presented in eTable 3 and eTable 4 in the Supplement. The pseudo *R*^2^ (estimated percentage of variance explained by all factors in the model) for change in trust and burnout explained 5% of the variance in burnout, and the pseudo *R*^2^ for change in trust and intent to leave explained 13% of the variance in intent to leave.

**Figure.**
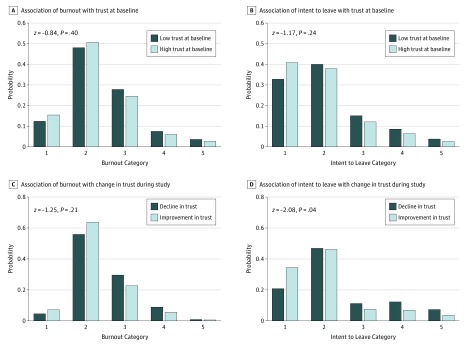
Multilevel Mixed-Effects Ordered Logistic Regression Model for the Association of Trust and Change in Trust With Burnout and Intent to Leave Burnout categories were scored by clinicians’ own definition of burnout and scored as 1 indicating “I enjoy my work. I have no symptoms of burnout”; 2, “Occasionally, I am under stress, and I don’t always have as much energy as I once did, but I don’t feel burned out”; 3, “I am definitely burning out and have one or more symptoms of burnout, such as physical and emotional exhaustion”; 4, “The symptoms of burnout that I’m experiencing won’t go away. I think about frustrations at work a lot”; and 5, “I feel completely burned out and often wonder if I can go on. I am at the point where I may need some changes or may need to seek some sort of help.” Intent to leave was classified as clinicians’ self-reported likelihood that they will leave their current practice in 2 years, with 1 indicating none; 2, slight; 3, moderate; 4, likely; and 5, definitely.

Separate regressions by sex did not disclose many meaningful sex differences, perhaps because of small sample sizes. *R*^2^ and pseudo *R*^2^ values were modest, ranging from 5% to 25%. The exception was for models on the association of improvement in trust with satisfaction among men (*R*^2^, 47%) and intent to leave among men (pseudo *R*^2^, 63%).

## Discussion

In this study of 165 clinicians across 34 practices, we found that clinicians’ trust in their organizations could be measured and tracked through time and was associated with a number of modifiable work conditions, including work control, cohesiveness, emphases on quality vs productivity and on communication, and values alignment with leadership. Some aspects of trust differed by sex. For example, men were more likely than women to express a high degree of organizational trust and loyalty to the organization. Clinician trust at baseline was associated with satisfaction. Additionally, compared with clinicians with decreased or lower trust, those in whom trust improved or remained high were more likely to have higher satisfaction, less stress, and less intention to leave their practice.

These findings are important as health care organizations aim to enhance professional satisfaction and improve patient outcomes in rapidly shifting health care environments. Our study has particular value, as it addresses a positive organizational aspect (ie, trust) as well as clinician satisfaction, with the 2 factors closely associated. Better knowledge of the associations and outcomes of these variables will allow organizations to target interventions to increase clinician satisfaction, retention, loyalty, and, hopefully, quality of patient care.^12^ Both within and outside health care,^19,20^ it is increasingly recognized that trust is an important component of well-functioning organizations, but measuring and promoting trust has been challenging; these data from the HWP study^11^ may provide a roadmap for doing so.

To our knowledge, little empirical work has focused on mediators and consequences of clinicians’ trust in their organizations. A 2004 study by Firth-Cozens^14^ suggested that clinician trust is associated with flatter hierarchies, open communication, teamwork, and empowered staff. Our study builds on a 2016 study by West et al^30^ by measuring trust across a diverse set of clinicians and practices using a validated measure of trust and characterizing the associations with work environment, physician characteristics, and professional satisfaction. We suggest that trust is associated with workplace conditions and thus is potentially modifiable. From a policy perspective, these findings provide a focus for organizational change as well as a direction for future studies to assess how patient trust and patient outcomes are associated with these favorable clinician findings.

Key organizational characteristics associated with trust included work control and organizational culture, including an emphasis on quality, communication, cohesiveness, and values alignment. These may be crucial characteristics to review for organizations that wish to build clinician trust. Lack of control of work is a well-described contributor to stress, yet organizations often remove clinician control by standardizing work. In our study, clinicians without control were substantially less likely to trust their organizations. Likewise, organizational cultures that favored quality, communication, cohesiveness, and values were all more likely to find trust among their clinicians. Addressing these cultural aspects are likely to produce environments where trust can flourish.

The domains that compose trust are also of interest. Loyalty, safety, belonging, and helping clinicians in need, along with a high sense of trust, compose clinician organizational trust. Understanding these domains would allow organizations to recognize what composes trust and begin to build it. Do clinicians feel loyal to their group? Is there a sense of belonging? If they are in trouble, is someone likely to help them? Knowing these dimensions will allow a clearer definition of what trust is based on and allow better modeling of how to advance it.

While all clinician outcomes were better in clinicians with high trust at baseline, only satisfaction was significantly higher. This provides a glimpse of how trust can promote joy in work,^31^ an emphasis among many health care organizations. While mechanisms to foster joy have been promoted, trust has not been among them, to our knowledge. Thus, trust could be explored in future trials as a means of improving satisfaction.

Of particular interest to many medical leaders may be our finding that improving trust was associated with improved satisfaction and had a significant association with decreased intention to leave one’s practice. According to estimates by the *New England Journal of Medicine* CareerCenter,^32^ Hamidi et al,^33^ and Schloss et al,^34^ the overall cost of physician turnover may be as high as $1 million per physician, including recruitment, onboarding, and lost revenue. There are also likely unmeasured consequences for patient satisfaction and practice stability.^35^ Interventions that enhance clinician trust may protect against these negative financial effects.

Our study highlights differences in trust by sex, with women less likely to express a high degree of trust or loyalty. These findings raise questions about whether medical practices are welcoming for women. Men continue to dominate most leadership roles, and there are well-documented disparities in pay and professional advancement for women.^36,37,38,39^ Medicine struggles with many of the societal issues that have commanded national attention; trust in organizations among women may be impaired until we make advances in these areas.

There were high percentages of variance in satisfaction and intent to leave associated with trust among men. Future research should explore interventions to create trust among all clinicians and determine the extent to which clinician trust is associated with cost, quality, and patient outcomes.^30^ Initiatives that promote best practices for engendering trust, such as that advanced by the American Board of Internal Medicine Foundation,^40^ may provide important insights into how organizations can build and repair trust when it is broken.

### Limitations

Our study has some limitations. First, we surveyed a relatively small number of clinicians; thus, many of the analyses may have missed significant effects. Second, the study was conducted at mainly academic primary care practices in the upper Midwest and East Coast. Although the clinics included rural, suburban, and inner-city sites, the clinicians may not be representative of those practicing in other care settings, subspecialties, or regions. Third, clinicians self-reported levels of trust, which is subject to biases, including recall bias and cognitive dissonance. Fourth, while we were able to identify associations with trust at baseline and with improved trust through time, we were not able to confirm causality. Thus, we view the study as mainly exploratory and encourage others to prospectively define interventions that can change trust in clinicians.

## Conclusions

The HWP study^11^ is among the first studies to examine workplace factors associated with clinician trust in their organizations and favorable outcomes associated with increasing trust, to our knowledge. In particular, clinicians in whom trust increased over time were more likely to have improvements in satisfaction, stress, and intention to leave. Overall, we found trust was associated with workplace control, cohesiveness, an emphasis on quality vs productivity and on communication, and values alignment with leadership. Practices hoping to promote clinician trust may find it useful to concentrate efforts in these areas.

## Notes

Supplement. **eTable 1.** Multilevel Assessment of Satisfaction and Stress by Trust at Baseline **eTable 2.** Multilevel Assessment of Satisfaction and Stress at Time 2 as a Function of Change in Trust **eTable 3.** Multilevel Ordered Logit Model of Burnout and Intention to Leave at Baseline Stratified by Trust **eTable 4.** Multilevel Ordered Logit Model of Burnout and Intention to Leave by Change in Trust
