# Error in Abstract, Text, and Table

**DOI:** 10.1001/jamanetworkopen.2019.9999

**Published:** 2019-08-02

In the Original Investigation titled “Characteristics of Health Care Organizations Associated With Clinician Trust: Results From the Healthy Work Place Study,”^1^ published June 21, 2019, the numbers of clinicians who worked in general internal medicine and family medicine were inadvertently transposed in the Abstract’s Results section, the first paragraph of the text’s Results section, and Table 1. Also in Table 1, a row of data for men was omitted and should have indicated 36 men (45.6%) with low trust and 43 (54.4%) with high trust. This article has been corrected.^1^
