# Supplementary material for: Characteristics of Health Care Organizations Associated With Clinician Trust: Results From the Healthy Work Place Study
Source: JAMA Netw Open. 2019 Jun 21;2(6):e196201. doi: 10.1001/jamanetworkopen.2019.6201 (PMC6593631; doi:10.1001/jamanetworkopen.2019.6201)
Supplement: Supplement. — eTable 1. Multilevel Assessment of Satisfaction and Stress by Trust at Baseline eTable 2. Multilevel Assessment of Satisfaction and Stress at Time 2 as a Function of Change in Trust eTable 3. Multilevel Ordered Logit Model of Burnout and Intention to Leave at Baseline Stratified by Trust eTable 4. Multilevel Ordered Logit Model of Burnout and Intention to Leave by Change in Trust [file jamanetwopen-2-e196201-s001.pdf]

Supplementary Online Content

Linzer M, Poplau S, Prasad K, et al; Healthy Work Place Investigators. Characteristics of health care organizations associated with clinician trust: results from the Healthy Work Place study. *JAMA Netw Open*. 2019;2(6):e196201. doi:10.1001/jamanetworkopen.2019.6201

eTable 1. Multilevel Assessment of Satisfaction and Stress by Trust at Baseline
eTable 2. Multilevel Assessment of Satisfaction and Stress at Time 2 as a Function of Change in Trust
eTable 3. Multilevel Ordered Logit Model of Burnout and Intention to Leave at Baseline Stratified by Trust
eTable 4. Multilevel Ordered Logit Model of Burnout and Intention to Leave by Change in Trust

This supplementary material has been provided by the authors to give readers additional information about their work.

© 2019 Linzer M et al. *JAMA Network Open.*

eTable 1. Multilevel Assessment of Satisfaction and Stress by Trust at Baseline

					95% Confidence Interval	
SATISFACTION	Coefficient	Std. Err.	Z-value	P>|z|	Lower	Upper
Fixed Coefficients						
Trust	.4871656	.1029409	4.73	0.000	.2854051	.6889261
Age	-.0012689	.0057556	-0.22	0.83	-.0125497	.0100119
Gender	-.0365287	.1036516	-0.35	0.73	-.2396821	.1666247
MD_NP	.3189099	.1582278	2.02	0.04	.0087891	.6290307
Specialty	.0672962	.1311296	0.51	0.61	-.1897132	.3243055
Intercept	3.530963	.2951159	11.96	0.000	2.952546	4.10938
Random coefficient						
Clinic Var	.0504033	.0364849			.0121986	.2082612
Clinician Var	.3685704	.0461601			.2883468	.4711137
Snijders/Bosker R-squared Clinician Level: 0.1213						
					95% Confidence Interval	
STRESS	Coefficient	Std. Err.	Z-value	P>|z|	Lower	Upper
Fixed Coefficients						
Trust	-.1704647	.120921	-1.41	0.16	-.4074674	.0665379
Age	.0069518	.006723	1.03	0.30	-.0062261	.0201297
Gender	.278974	.1278975	2.18	0.03	.0282996	.5296484
MD_NP	-.0630937	.1893996	-0.33	0.74	-.4343102	.3081228
Specialty	-.1823155	.130193	-1.40	0.16	-.4374891	.0728581
Intercept	3.052764	.3473498	8.79	0.002	2.371971	3.733557
Random coefficient						
Clinic Var	1.25e-21	1.13e-20			2.69e-29	5.84e-14
Clinician Var	.5826403	.0641466			.4695548	.7229607
Snijders/Bosker R-squared Clinician Level: 0.0565						

© 2019 Linzer M et al. *JAMA Network Open.*

eTable 2. Multilevel Assessment of Satisfaction and Stress at Time 2 as a Function of Change in Trust

					95% Confidence Interval	
SATISFACTION	Coefficient	Std. Err.	Z-value	P>|z|	Lower	Upper
Fixed Coefficients						
Change in Trust	.5562188	.0984534	5.65	0.000	.3632538	.7491839
Age	-.0063549	.0052603	-1.21	0.23	-.0166648	.003955
Gender	-.0810343	.0995766	-0.81	0.42	-.2762008	.1141323
MD_NP	.0599298	.1483781	0.40	0.69	-.2308859	.3507455
Specialty	.0131251	.1096511	0.12	0.91	-.2017871	.2280373
Intercept	3.779622	.267927	14.11	0.000	3.254495	4.30475
Random coefficient						
Clinic Var	.0113747	.0190684			.0004256	.3040039
Clinician Var	.270392	.03708			.206664	.3537714
Snijders/Bosker R-squared Clinician Level 0.2271						
					95% Confidence Interval	
STRESS	Coefficient	Std. Err.	Z-value	P>|z|	Lower	Upper
Fixed Coefficients						
Change in Trust	-.2919311	.1354968	-2.15	0.03	-.5574999	-.0263622
Age	-.0013935	.0073298	-0.19	0.85	-.0157597	.0129726
Gender	.2501225	.1418222	1.76	0.08	-.0278439	.528089
MD_NP	.0863091	.2092902	0.41	0.68	-.3238921	.4965104
Specialty	-.168139	.1447054	-1.16	0.25	-.4517563	.1154783
Intercept	3.487392	.3745352	9.31	0.000	2.753317	4.221468
Random coefficient						
Clinic Var	1.13e-17	8.65e-17			3.71e-24	3.47e-11
Clinician Var	.5591286	.0693514			.4384632	.7130013
Snijders/Bosker R-squared Clinician Level: 0.0899						

© 2019 Linzer M et al. *JAMA Network Open.*

eTable 3. Multilevel Ordered Logit Model of Burnout and Intention to Leave at Baseline Stratified by Trust

					95% Confidence Interval	
BURNOUT	Coefficient	Std. Err.	Z-value	P>|z|	Lower	Upper
Fixed Coefficients						
Trust	-.2728107	.325615	-0.84	0.40	-.9110044	.365383
Age	.0040104	.0187273	0.21	0.83	-.0326943	.0407152
Gender	.7065823	.3424426	2.06	0.04	.0354072	1.377757
MD_NP	-.3191949	.5004943	-0.64	0.52	-1.300146	.661756
Specialty	-.173997	.4522976	-0.38	0.70	-1.060484	.71249
Threshold1	-1.711753	.9834808			-3.63934	.2158343
Threshold2	.9502575	.9683743			-.9477212	2.848236
Threshold3	2.741871	1.003326			.7753879	4.708353
Threshold4	4.014132	1.072769			1.911542	6.116721
Random coefficient						
var(_cons)	.5105468	.467857			.0847243	3.076545
McKelvey&Zavoina-Pseudo-R2 (fixed & random effects) = 0.0794						
					95% Confidence Interval	
INTENT TO LEAVE	Coefficient	Std. Err.	Z-value	P>|z|	Lower	Upper
Fixed Coefficients						
Trust	-.3710462	.3178411	-1.17	0.24	-.9940032	.2519109
Age	.0257887	.019079	1.35	0.18	-.0116054	.0631828
Gender	.2595195	.3238944	0.80	0.42	-.3753019	.8943409
MD_NP	-.0554302	.5089863	-0.11	0.91	-1.053025	.9421646
Specialty	-.3930586	.3820733	-1.03	0.30	-1.141909	.3557914
Threshold1	.429451	.9384849			-1.409946	2.268848
Threshold2	2.246933	.971339			.3431437	4.150723
Threshold3	3.287563	1.004096			1.319571	5.255555
Threshold4	4.607085	1.080597			2.489154	6.725016
Random coefficient						
var(_cons)	.2963161	.2863364			.0445883	1.969196
McKelvey&Zavoina-Pseudo-R2 (fixed & random effects) = 0.0489						

© 2019 Linzer M et al. *JAMA Network Open.*

eTable 4. Multilevel Ordered Logit Model of Burnout and Intention to Leave by Change in Trust

					95% Confidence Interval	
BURNOUT	Coefficient	Std. Err.	Z-value	P>|z|	Lower	Upper
Fixed Coefficients						
Change in Trust	-.4918845	.393937	-1.25	0.21	-1.263987	.2802179
Age	.0093725	.021368	0.44	0.66	-.032508	.0512529
Gender	.5461799	.3935068	1.39	0.17	-.2250792	1.317439
MD_NP	.0460746	.5735918	0.08	0.94	-1.078145	1.170294
Specialty	-.4198128	.4633771	-0.91	0.37	-1.328015	.4883897
Threshold1	-2.568023	1.084542			-4.693686	-.4423602
Threshold2	1.038905	1.064871			-1.048204	3.126014
Threshold3	2.90873	1.122098			.7094583	5.108002
Threshold4	5.316942	1.480986			2.414263	8.21962
Random coefficient						
var(_cons)	.1515186	.3422952			.0018093	12.68857
McKelvey&Zavoina-Pseudo-R2 (fixed & random effects) = 0.0543						
					95% Confidence Interval	
INTENT TO LEAVE	Coefficient	Std. Err.	Z-value	P>|z|	Lower	Upper
Fixed Coefficients						
Change in Trust	-.7318086	.3511253	-2.08	0.04	-1.420001	-.0436157
Age	.0549693	.0196635	2.80	0.01	.0164297	.093509
Gender	.7282461	.3701235	1.97	0.05	.0028173	1.453675
MD_NP	.0470185	.5485341	0.09	0.93	-1.028089	1.122126
Specialty	-.2442437	.3671837	-0.67	0.51	-.9639105	.475423
Threshold1	1.483973	.9997864			-.4755718	3.443519
Threshold2	3.720357	1.046724			1.668816	5.771897
Threshold3	4.371057	1.065662			2.282398	6.459715
Threshold4	5.534355	1.12435			3.330668	7.738041
Random coefficient						
var(_cons)	1.97e-30	2.57e-15			.	.
McKelvey&Zavoina-Pseudo-R2 (fixed & random effects) = 0.1311						

© 2019 Linzer M et al. *JAMA Network Open.*
